# Progress on Optical Fiber Biochemical Sensors Based on Graphene

**DOI:** 10.3390/mi13030348

**Published:** 2022-02-23

**Authors:** Yani Zhang, Lei Zhou, Dun Qiao, Mengyin Liu, Hongyan Yang, Cheng Meng, Ting Miao, Jia Xue, Yiming Yao

**Affiliations:** 1 Department of Physics, School of Arts & Sciences, Shaanxi University of Science & Technology, Xi’an 710021, China; 200911070@sust.edu.cn (T.M.); 210911048@sust.edu.cn (J.X.); 210911040@sust.edu.cn (Y.Y.); 2 School of Electrical and Control Engineering, Shaanxi University of Science & Technology, Xi’an 710021, China; 200611002@sust.edu.cn (L.Z.); 200612080@sust.edu.cn (C.M.); 3 Faculty of Computing, Engineering and Science, Wireless and Optoelectronics Research and Innovation Centre, University of South Wales, Pontypridd CF37 1DL, UK; dun.qiao@southwales.ac.uk; 4 Photonics Research Center, School of Optoelectronic Engineering, Guilin University of Electronic Technology, Guilin 541004, China; 19082203009@mails.guet.edu.cn; 5 Guangxi Key Laboratory of Automatic Detecting Technology and Instruments, Guilin University of Electronic Technology, Guilin 541004, China

**Keywords:** graphene, optical fiber, biochemical sensor, photonic crystal fiber, fiber grating, evanescent wave

## Abstract

Graphene, a novel form of the hexagonal honeycomb two-dimensional carbon-based structural material with a zero-band gap and ultra-high specific surface area, has unique optoelectronic capabilities, promising a suitable basis for its application in the field of optical fiber sensing. Graphene optical fiber sensing has also been a hotspot in cross-research in biology, materials, medicine, and micro-nano devices in recent years, owing to prospective benefits, such as high sensitivity, small size, and strong anti-electromagnetic interference capability and so on. Here, the progress of optical fiber biochemical sensors based on graphene is reviewed. The fabrication of graphene materials and the sensing mechanism of the graphene-based optical fiber sensor are described. The typical research works of graphene-based optical fiber biochemical sensor, such as long-period fiber grating, Bragg fiber grating, no-core fiber and photonic crystal fiber are introduced, respectively. Finally, prospects for graphene-based optical fiber biochemical sensing technology will also be covered, which will provide an important reference for the development of graphene-based optical fiber biochemical sensors.

## 1. Introduction

Graphene is a two-dimensional single-crystal functional material with a hexagonal honeycomb lattice composed of carbon atoms with sp^2^ hybrid orbitals, and has been considered a hypothetical structure for a long time. In 2004, graphene was separated by the 2010 Nobel Prize winners Andre Geim and Konstantin Novoselov [1], who made it become a distinct entity. Three in-plane σ bonds and one out-of-plane π bond exist in each carbon atom of graphene and are responsible for out-of-plane bonding, such as the stacking of graphene sheets. [2]. The carbons inside the monolayer graphene sheet are connected by covalent bonds, while the different graphene layers are connected by van der Waals bonds. Monolayer graphene has an extremely high surface-to-volume ratio due to the ultra-thin thickness (~0.3 nm). [3]. Moreover, graphene has a very active surface area, as all the carbon atoms are located on the surface. In addition, it also has outstanding mechanical, chemical and op-tical properties. [4]. Graphene is quite sensitive to the external environment because each atom of graphene is a surface atom; therefore, graphene-based nanostructures have great potential for developing various types of sensors [5]. Because of these characteristics, in less than 20 years, graphene has been widely studied in many different fields, such as field emission displays, transparent conductors, field effect transistors (FETs), optical biochemical sensors and composite reinforcement of material, etc. [6,7,8,9,10,11,12,13,14,15,16].

Optical biochemical sensors have broad development prospects because of their advantages of non-destructive detection, high sensitivity and fast detection speed, etc. [17]. The optical fiber sensor, comprised of an important branch of optical sensors and integrating signal acquisition and transmission, has become a research hotspot in the field of sensing. In the past ten years, optical fiber-based biochemical sensors have attracted people’s interest due to their attractive features such as compact size, flexibility, relatively low cost, and biocompatibility [18]. Additionally, their magnetic resonance compatibility and distant and multiplexed detection capabilities [19] allow them to be used in a variety of industries [20]. The physical properties of optical fibers make it possible to measure the level of biomarkers and chemical markers in biological and chemical liquids, non-liquid media, and hard-to-reach environments for minimally invasive in vivo medical diagnosis [21]. The sensing mechanism of the fiber-optic biochemical sensor modulates the physical signal of the transmitted light in the fiber, such as refractive index (RI), intensity, amplitude and phase by using the biochemical information, which is generated by the selective interaction (i.e., specific binding of antigen-antibody or receptor-ligand; nucleic acid molecular base complementary pairing; specificity of enzyme to substrate, etc.) between the measured object and the biological sensor. Among them, the biochemical sensitive element is the key component of the sensor, and the general biochemical sensitive element is comprised of antigens, antibodies, enzymes, or oligonucleotides [22,23,24,25]. In order to improve the sensitivity of biochemical detection, the contact area between light and the biochemical sensitive element is increased by changing the optical fiber structure at a specific detection point or covering the sensitive medium material at the end of the optical fiber. Then, the transmission spectrum or reflection spectrum caused by the change of the surrounding biochemical sensitive mediumis detected by a spectral analyzer or vector analyzer [26].

As a new functional 2D material, graphene can be easily integrated into optical fiber because of its high electrical conductivity, flexibility, strength, and lack of dangling bonds. Then, graphene has no mass and has an ultra-fast migration speed (up to 200,000 cm^2^/Vs) at room temperature, a high specific surface area and a unique Dirac cone structure leading to zero-band gaps. All these contribute to absorption on the surface of biomolecules, leading to extremely sensitive biomolecule sensing. In recent years, graphene biochemical sensors have been explored by numerous scholars due to their obvious advantages, high sensitivity, and label-free, real-time detection of bio-logical molecules (protein and DNA, etc.) [27,28,29]. For example, Phitsini S. et al. in 2017 reviewed the possibilities of graphene biosensing based on the intrinsic properties of graphene and its surface sensitivity concerned with the transduction mechanisms in biosensing applications [30]. In 2017, Corina A. et al. reviewed the research of graphene-based field effect transistors biosensors by using the electrical properties of graphene [31]. In 2021, Novodchuk I. et al. reviewed further graphene-based field effect transistor biosensors for breast cancer detection [4]. In 2019, Li Zongwen et al. reviewed the graphene-based optical biosensors to detect the single cell, cell line (e.g., HCT116 and LoVo), anticancer drug, protein and antigen–antibody based on the unique optical properties of graphene [32]. In 2017, Miguel H. et al. reviewed the graphene-based optical fiber sensors by combining the chemical, optical, and electrical features of graphene with the advantages of optical fiber [33]. Moreover, In 2021, AN Ning et al. reviewed the emerging graphene-fiber biochemical sensors from combining the merits of both graphene and fiber structures [34]. Throughout the reviews mentioned above, the combination of graphene and optical fiber includes covering the outer surface of the micro-nano fiber, replacing the cladding of fiber, filling in the air hole of photonic crystal fiber (PCF), and polishing the side surface of D-type fiber. There are still many typical graphene-based fiber biochemical sensors combining fiber structure changes that have not been mentioned, such as long-period gratings, Bragg gratings, coreless fibers and special photonic crystal fibers.

In this paper, development of optical fiber biochemical sensors based on graphene is reviewed. Firstly, four basic methods of preparing graphene, including mechanical the exfoliation method, chemical vapor deposition method, epitaxial growth method, and oxidation-reduction method are introduced, and the advantages and disadvantages of each method are stated. The next section describes the graphene-based optical fiber-sensing mechanism, including sensing properties of graphene and the graphene optical fiber-sensing mechanism. In the following sections, we review recent progresses in different various typical types of optical fiber biochemical sensors, covering the long-period fiber-grating sensor, Bragg fiber-grating sensor, no-core fiber sensor, and photonic crystal fiber sensor. Finally, the issues of the graphene optical fiber biochemical sensor are proposed and the progress to be made in the future is described, which not only clearly clarifies the current development stage, but also provides an efficient feasible direction and guidance for the future exploration of graphene-based op-tical fiber biochemical sensors.

## 2. Graphene Synthesis Methods

Graphene, as one of the 2D-layered nanomaterials, can be synthesized in two techniques: top-down exfoliation and bottom-up growth. The top-down method, including the mechanical exfoliation method, liquid phase exfoliation technique and oxidation-reduction method (redox), is used to prepare single or few-layer 2D nanomaterials by destroying the van der Waals forces between the layers of 2D materials. The bottom-up method is the synthesis of 2D nanomaterials at the molecular level by chemical means, including chemical vapor deposition, epitaxial growth method, hydrothermal method, and pulsed magnetron sputtering, pulsed laser deposition, etc. [35,36,37,38,39,40]. Researchers synthesized a variety of layered materials by using these methods. For the application of optical fiber sensors, graphene is expected to have large area, uniform thickness, and smooth surface. Here, we made a detailed introduction on two common methods from each of the synthesis methods.

### 2.1. Mechanical Exfoliation Method

**Figure 1:**
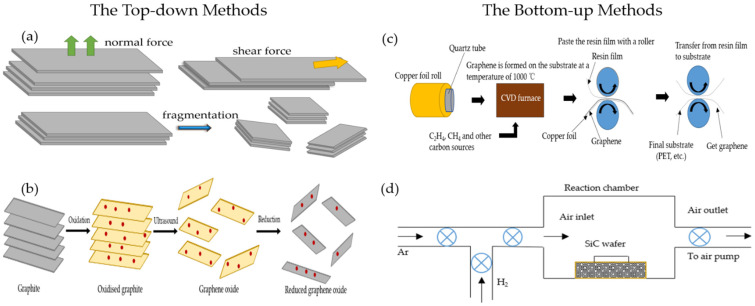
Synthesis process diagram of four different methods for preparing graphene (**a**) mechanical exfoliation method. (**b**) Oxidation-reduction method. (**c**) Chemical vapor deposition method. (**d**) Epitaxial growth method.

The mechanical exfoliation method, also known as the micromechanical cleavage technique, is an effective way to exfoliate graphene into single- or few-layer nanomaterials by applying mechanical force on layered bulk crystals. Figure 1a is a process flow diagram for preparing graphene by the mechanical exfoliation method. In 2004, Novoselov et al. successfully exfoliated the monolayer graphene in the highly oriented pyrolytic graphene by the micromechanical exfoliation method [1]. The independent existence of monolayer graphene was verified and measured, which performed many excellent optical, electrical, and thermal properties. While this method has the ability to synthesize high-quality multilayer or monolayer graphene, it is not suitable for large-scale application because of poor scalability and low production efficiency. In addition, the chemical traces left after the tape stripping are another issue to be addressed.

### 2.2. Oxidation-Reduction (Redox) Method

At present, the redox method is a relatively commonly used method for preparing graphene [41]. Figure 1b is the synthesis process of graphene preparation by the redox method. Firstly, the crystal structure of graphite is destroyed by a strong acid and oxidant to generate graphite oxide. Then, the graphite oxide is separated by ultrasonic separation to form graphene oxide. Finally, graphene is obtained by adding a reducing agent to remove oxygen-containing groups on the surface of the graphene-oxide (GO), such as carboxyl, epoxy, and hydroxyl [42]. The Staudenmaier method [43], Brodie method [44], and Hummers method [45] are the three main methods for the preparation of GO. Song et al. [46] firstly used the modified Hummers method to oxidize graphite to produce GO through H_2_SO_4_, NaNO_3,_ and KMnO_4_. Then, reduction can obtain graphene by using hydrazine hydrate and ammonia water as reducing agents and mixing the reducing agent with the GO solution by ultrasonic dispersion. Redox has the advantages of low environmental pollution, high efficiency, high feasibility, low cost, and large-scale production in the industry, etc. However, it is easy to produce the pollution of liquid waste during the synthesis process and to destroy the electronic structure of the graphene crystal due to strong oxidizers, which affects the performance of graphene and limits its development in the field of microelectronics.

### 2.3. Chemical Vapor Deposition (CVD) Method

CVD synthesizes graphene by the chemical decomposition material of carbon source under a certain temperature or in an external field and deposition on the substrate surface [47,48,49]; its process diagram is shown in Figure 1c. CVD system includes: the gas conveying system, reaction chamber, and exhaust system. The reaction process consists of heating up, heat treatment of substrate, graphene growth, and cooling of graphene. Here, carbon sources can be gaseous hydrocarbons (such as methane and ethylene etc.), liquid (such as ethanol and benzene, etc.), or solid (such as PMMA and amorphous carbon, etc.) [47]. Reaction substrates are generally divided into metal (such as copper and nickel, platinum, etc.) [50,51,52] and non-metal (such as silicon oxide and silicon nitride, glass, etc.). Most of the graphene used in sensors is made by the CVD method, because CVD offers a scalable method for the production of graphene that has a big area and is easy to transfer with little pollution.

### 2.4. Epitaxial Growth Method

In 2006, Bergeretal C. et al. used the epitaxial growth method to prepare monolayer and multilayer graphene sheets [53]. The thickness of the graphene sheets is adjusting by the heating temperature. According to the different substrate material, the epitaxial growth methods are categorized as the silicon carbide (SiC) epitaxial growth [54] and metal-catalyzed epitaxial growth [55]. Here, the process diagram of the SiC epitaxial growth is shown in Figure 1d. the SiC epitaxial growth method [56] is heating SiC to a certain temperature under a certain vacuum, and causing the silicon atoms to evaporate and the remaining carbon atoms to form graphene based on the SiC substrate. The prepared graphene has the advantages of good uniformity, large area, and high quality, etc. However, this method requires a high-temperature and vacuum environment, and the produced graphene is difficult to separate from the substrate, therefore, it cannot be used in mass production.

## 3. The Sensing Properties and Mechanism of Graphene-Based Optical Fiber

Graphene has been considered as a multifunctional material because of its 2D planar structure. It has excellent potential applications in wide range of fields, such as photonics, optoelectronics, sensors, flexible electronics and nanocomposites, etc. [57]. In particular, the integration of graphene and its derivatives with biochemical sensor modules also can also be applied to detect different samples, such as cells, proteins, and small molecules [58,59]. The chemical derivatives of graphene, including graphene-oxide (GO), reduced-GO (rGO), few-layer graphene (FLG), wrinkled graphene (WG), hydrogenated graphene (HG), and nano-size GO etc. are also broadly applied as a components for biosensors and cancer diagnosis and treatment, etc. [60,61]. Here, the biochemical-sensing mechanism of graphene-based optical fiber was classified and analyzed based on the unique photoelectric properties of graphene.

### 3.1. Sensing Properties of Graphene

**Figure 2:**
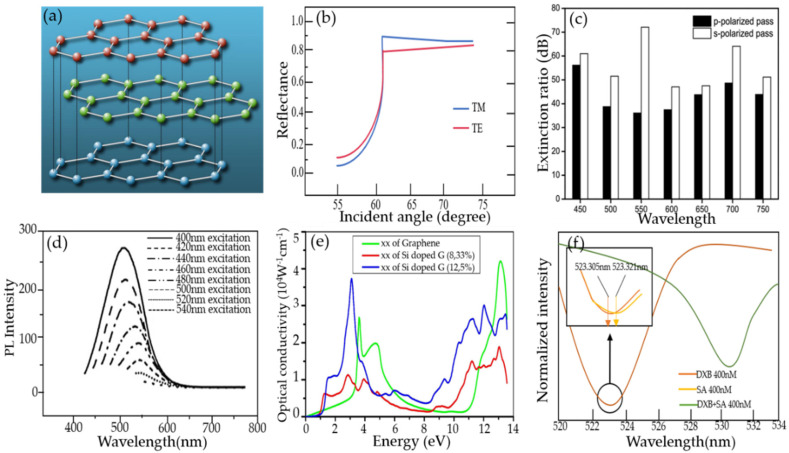
(**a**) The structure of graphene. (**b**) Experimental and calculated angular dependence of the ratio of optical reflectance of TM wave to monolayer TE wave. (**c**) Extinction ratio at different wavelengths in s- and p-polarized pass situation. (**d**) Variation of GQDs excitation wavelength. (**e**) Optical conductivity of the monolayer graphene up to ultraviolet frequency. (**f**) SPR-sensing characteristics. Reproduced from [67,77,78] with permission of the Elsevier.

The structure of graphene is shown in Figure 2a. The various properties of graphene are excellent: its strength is about 200 times that of steel, its electrical and thermal conductivity are higher than copper, and its weight per square meter is less than 1 milligram. The reason why graphene has high electrical conductivity and thermal conductivityis that its room temperature electron mobility is as high as 15,000 cm^2^/(Vs). Because of its excellent carrier mobility and mechanical properties, graphene is widely favored by researchers in condensed matter physics and materials science. Graphene has been widely used in the field of biochemical sensing based on its good photoelectric characteristics and excellent high-energy transfer efficiency, large surface area, and biocompatibility [62]. This section focuses on four important properties of graphene in improving the sensitivity of fiber-optic biochemical sensors, including optical adsorption properties, optical conductivity, photoluminescence properties and surface plasmon properties.

#### 3.1.1. Optical Absorption Characteristics

Graphene has excellent optical absorption characteristics. The absorption rate of monolayer graphene to visible light is only 2.3% [63], and the reflectivity is negligible. The light absorptivity of graphene increases linearly with the graphene layers. The theoretical research indicated that monolayer, double-layer, and few-layer graphene absorb the TE (transversal electric wave) mode more than TM (transversal magnetic wave) mode in the total internal reflection structure [64]. As shown in Figure 2b, the different light absorptivity of graphene leads to the reflectivity of the TM mode, which is greater than the TE mode [65]. Therefore, the resolution and sensitivity of the sensing system can be greatly improved by measuring the difference in the reflected light intensity between the TM mode and TE mode. Graphene exhibits strong polarization-dependent optical absorption under total internal reflection [66]. Figure 2c shows that the extinction ratios of the graphene polarizer at different wavelength in s- and p-polarized passing situation [67]. All of the extinction ratios are higher than 36 dB, which means that the graphene polarizer has an excellent polarization performance. For optical fiber sensors, the light absorption performance of graphene can greatly improve the sensing resolution. Therefore, improving and enhancing the light absorption properties of graphene by adopting various new technological methods has become a pursuit of researchers. [68,69,70,71,72].

#### 3.1.2. Photoluminescence Characteristics

Photoluminescence (PL) is an optical phenomenon that semiconductors launch light emissions by absorbing incident light, in which semiconductors emit light by absorbing incident light with energy higher than the energy band gap of the semiconductor. In the mechanism of PL, the excited electrons generated by optical excitation will return to the ground state with the emission of photons [73]. Graphene is a photoluminescent material. Graphene quantum dots (GQDs) are generally regarded as a new material with graphene sheet sizes less than 100 nm, the number of graphene sheet layers is less than 10. GQDs can produce a photoluminescence effect, which is the most important and most widely studied performance of graphene, and also makes graphene close to practical application [74]. As shown in Figure 2d, GODs generate different fluorescence through different excitation wavelengths; the light with a wavelength of 400 nm to 540 nm can excite green fluorescence, and the fluorescence intensity is reduced and the fluorescence peak will have a red shift with the increase of the excitation wavelength. Because of this, graphene is widely used in biochemical detection, such as bioimaging, optical sensing, biochemical sensing and therapy [75,76].

#### 3.1.3. Optical Conductivity

Graphene’s unique structure enables it to have special properties, such as the perfect quantum tunneling effect and quantum Hall effect, etc. The optical conductivity of graphene depends on electron density, layer number and width at low temperatures. When electrons are transmitted in the graphene layer, the interference is very small and it is not easy to be scattered. The mobility can reach 2 × 10^5^ cm^2^/(V·s), which is about 100 times that of the electron in silicon and the resistivity is about 10^−6^ Ω·cm, which is lower than that of metallic copper or silver. Therefore, it is the material with the lowest resistance at room temperature, and its conductive density is one million times that of copper. As the number of graphene layers increases, its conductivity gradually decreases, and then tends to remain unchanged at eight monolayers [79]. The optical conductivity of monolayer graphene is proportional to the number of layers when the light energy is larger than twice the kinetic energy of the Fermi level. Doped graphene will also increase its optical conductivity. Figure 2e shows the optical conductivity of graphene with and without silicon doping [78]; the optical conductivity of silicon-doped graphene is higher than that of pure graphene, with a peak of 2.66 eV for silicon-doped graphene and 3.78 eV for graphene because the optical conductivity is related to the absorption spectrum.

#### 3.1.4. Surface Plasmon Properties

Graphene is an excellent plasmon material [77], which is applied to optical fiber sensors by the surface plasmon effect to increase the sensitivity of biochemical sensors. As shown in Figure 2f, the graphene-coated fiber forms a surface plasmon resonance (SPR) structure as surface plasmon. The resonant wavelength of SPR will change with the refractive index of biochemical analytes. The common materials to excite surface plasmon are noble metals, such as gold or silver. However, due to the existence of noble metal resistance, its large loss in visible light band and the complexity of metal coating process make it very easy to be oxidized and worn; therefore, the sensitivity of its sensing is greatly limited. Graphene has stable chemical properties, is not easily oxidized, and can prevent metal oxidation, increase the elastic modulus of metal surface, and promote the effective propagation of surface plasma in the infrared band. Therefore, surface plasmon-graphene can improve the sensitivity of fiber optic biochemical sensors, and has been widely used in the field of biochemical sensing [77,80].

### 3.2. The Sensing Mechanism of Graphene-Based Optical Fiber

The energy band of graphene has a linear dispersion relation, and its Dirac cone structure provides a unique zero band gap, large specific surface area, ultra-broadband optical spectrum, and strong nonlinear optical properties, which enable it to have a certain optical transmission ability. In this section, we will mainly introduce the energy band theory and sensing mechanism of graphene.

#### 3.2.1. Kubo Model of Graphene

**Figure 3:**
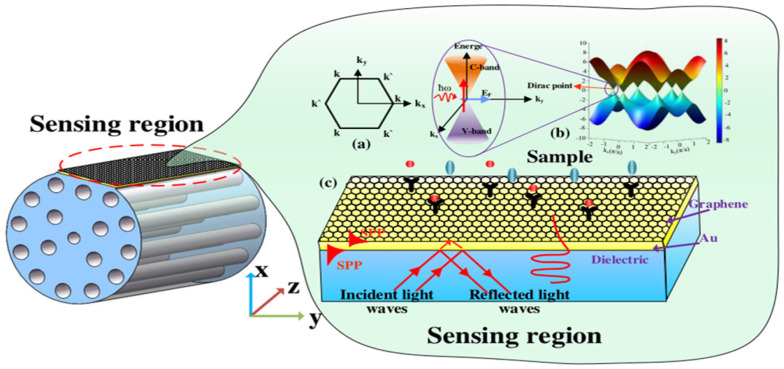
SPR-sensing mechanism of D-type photonic crystal fiber (PCF) sensor. (**a**) Graphene Brillouin zone. (**b**) Linear dispersion diagram of the band structure of monolayer graphene. (**c**) Schematic diagram of graphene-Au-sensing mechanism. Reproduced from [81] with permission of the Multidisciplinary Digital Publishing Institute.

It can be observed from Figure 3a that graphene is a two-dimensional honeycomb lattice structure material that is tightly packed with a monolayer of carbon atoms on the sp_2_ hybrid orbital. K_x_ and K_y_ are the components of the wave vector k. As k and k’ are symmetric, and the conduction and valence bands at two points in the Brillouin zone are degenerate, the linear dispersion relationship of the energy bands of graphene is obtained. Hence, electrons can be considered to be massless relativistic particles, namely Dirac fermions, and the dispersion relation of the two-dimensional electron energy is isotropic, and is called Dirac cones. [81,82]. Figure 3b shows the energy band structure of graphene, and the Dirac point region is enlarged in the inset. It can be observed that graphene is a semi-metallic material with zero-band gap. The conduction band (c band) and the valence band (v band) are symmetrically tapered and intersect at one point. This band structure satisfies the Dirac equation, rather than the Schrodinger equation satisfied by traditional metals or semiconductors, and the intersection is called the Dirac point. The unique zero bandgap, excellent mechanical properties, high thermal conductivity, large specific surface area, ultra-broad photo-response spectrum, and strong nonlinear optical properties have significant advantages in novel optical and optoelectronic-sensing technologies. Graphene can be transformed from semi-metallic to metallic behavior by chemical doping or electrical gating, which depends heavily on µ (chemical potential). [83,84,85,86]. From the complex Kubo equation, a random phase approximation of the dynamic optical response of graphene can be obtained, including inter-band and intra-band contributions [87,88].

(1) $$\sigma =\sigma_{\mathrm{intra}}+{\sigma^{\prime }}_{\mathrm{inter}}+{{i\sigma }^{\prime \prime }}_{\mathrm{inter}}$$

(2) $$\sigma_{\mathrm{intra}}=\sigma_{0}\frac{4\mu }{\pi }\frac{1}{\hbar \tau_{1}-i\hbar \omega }$$

Among them, σ_0_ = πe^2^/(2ℏ), τ_1_ is the intra-band transition relaxation rate, ℏ is the reduced Planck’s constant, µ > 0 is the chemical potential, and ω is the radian frequency. The inter-band contribution is:

(3) $${\sigma^{\prime }}_{\mathrm{inter}}=\sigma_{0}\left(1+\frac{1}{\pi }\arctan\frac{\hbar \omega -2\mu }{\hbar \tau^{2}}-\frac{1}{\pi }\arctan\frac{\hbar \omega +2\mu }{\hbar \tau^{2}}\right)$$

(4) $${\sigma^{\prime \prime }}_{\mathrm{inter}}=-\sigma_{0}\frac{1}{2\pi }\ln\frac{{\left(2\mu +\hbar \omega \right)}^{2}+\hbar^{2}\tau_{2}{}^{2}}{{\left(2\mu -\hbar \omega \right)}^{2}+\hbar^{2}\tau_{2}{}^{2}}$$

where τ_2_ is the inter-band transitions rate of relaxation.

From the above formular, it can be observed that the intra-band and inter-band optical conductivities of graphene are concerned with μ and ω. The μ of doped graphene is controlled by the carrier concentration n_0_ = (μ/hυ)^2^/π, which can be controlled by chemical doping or applied voltage. There is no intra-band contribution in the µ = 0 of pristine graphene. It can be observed from the theoretical expression and experimental results of optical absorption that the intra-band photoconductivity σ in the terahertz and far-infrared bands dominates due to the appearance of the zero-band gap, while the total conductivity variation in the near-infrared and visible light bands mainly depends on the transition process between the bands. Particularly, the propagation of surface plasmons in graphene has relations to the intra-band contribution.

#### 3.2.2. Sensing Mechanism-SPR and Evanescent Field

Figure 3c is the schematic diagram of SPR. The evanescent wave generated by the total reflection enters the metal layer through the interface between the metal and the medium, and interacts with free electrons to excite surface plasmon waves (SPWs) that propagate on the surface of the metal layer. SPR is a type of optical physical phenomenon. When the incident wavelength reaches a certain value (resonance), most of the incident light is converted into the energy of SPW, resulting in a sharp drop in the reflected light energy and a resonance peak in the reflection spectrum. The incident wavelength at this time is called the resonance wavelength of the SPR. By measuring the shift of the resonance wavelength, the refractive index (RI) of the sample on the surface of the metal layer can be obtained. [89].

The optical fiber evanescent wave biochemical sensor works based on the principle of the evanescent wave [90]. The evanescent wave in the optical fiber is generated when the light is transmitted between the core and the cladding through total internal reflection. The properties and content of biochemical substances within the evanescent wave field attached to the surface of the fiber core through a specific biochemical reaction are detected by exciting fluorescent dyes marked on molecules on the surface of the fiber core. This type of biochemical sensor is easy to operate, fast in measurement, short in time, high in sensitivity and specificity, and can be used for on-site detection and real-time dynamic monitoring of biological reactions. Evanescent wave sensors often use metal thin metal films as dielectric materials. However, the poor biocompatibility and instability of thin metal films has become a major obstacle to its development. The fiber-optic evanescent wave sensor with graphene as an absorption-enhancing layer to measure the hemoglobin concentration in human blood is proposed in 2018 by Sharma AK et al. [91]. A large number of researchers are attracted and involved in the research of the graphene fiber evanescent wave biochemical sensor due to the emergence of graphene.

## 4. Progress of Graphene Optical Fiber Biochemical Sensor

**Figure 4:**
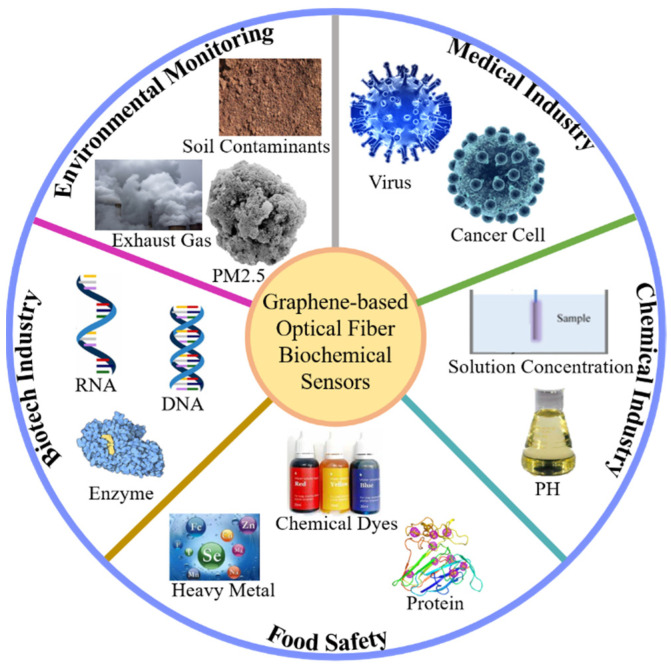
Diagrams of biochemical sensors for detecting important substances in different fields.

It is well known that biochemical sensors have been widely used in medical diagnosis, environmental monitoring, food safety, genetic engineering and other fields, and the accuracy and requirements of detection are becoming higher and higher with the development of artificial intelligence technology. Graphene and its derivatives (graphene oxide-GO), as new sensitive materials, are rapidly becoming a popular material for fiber-optic biochemical sensors. Based on the two-dimensional monolayer honeycomb lattice structure, GO is formed by enriching oxygen-containing functional groups (hydroxyl and carboxyl groups) on the surface of graphene through oxidation. Compared with graphene, the functional groups of GO are highly dispersed, hydrophilic and modifiable. Its unique two-dimensional structure and abundant oxy-gen-containing functional groups on the surface have presented unique advantages in biochemical-sensing applications. As shown in Figure 4, the combination of graphene and its derivatives with fiber-optic biochemical sensors have a wide range of applications in different fields. Recently, there have been many reviews on the applications of graphene sensors, including graphene field effect tube sensors, graphene gas sensors, and graphene surface plasma sensors, etc. However, the combination of special fiber with graphene to obtain highly sensitive fiber biochemical sensing has not been systematically reported. The following is a summary of the development of graphene and typical fiber biochemical sensors, including long-period grating, Bragg grating, coreless fiber, and photonic crystal fiber, etc.

### 4.1. Graphene Fiber-Grating Sensor

In recent years, researchers have discovered that the change in the effective refractive index of the optical fiber evanescent field caused by the combination of the substance to be tested; and the molecular film can be used to achieve detection, which involves using ultraviolet light to modify the refractive index of the fiber core on a regular basis in order to create a spatial phase grating [92]. Fiber grating is an intrinsic optical fiber sensing element, and small changes in the objective parameters of the surrounding environment of the grating will cause a wavelength shift, which also has biophilic activity and reusability, and the production process is relatively mature. Therefore, the fiber-grating biochemical sensors have quickly become one of the research hotspots in the field of biochemical sensing.

#### 4.1.1. Graphene Long-Period Fiber-Grating Sensor

**Figure 5:**
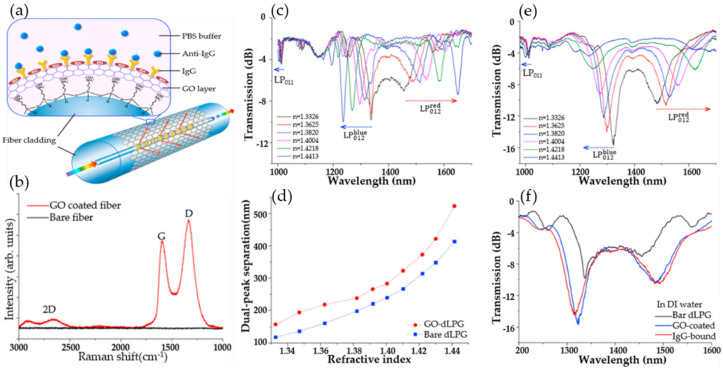
(**a**) Schematic diagram of optical fiber biosensor comprising the dLPG coated with the graphene oxide-linking layer. (**b**) The Raman spectrum of GO-coated fiber compared with bare fiber. (**c**) Transmission spectra of bare dLPG measured in different sucrose concentrations. (**d**) Dual-peak wavelength separation against SRI. (**e**) Transmission spectra of bare dLPG measured in different sucrose concentrations. (**f**) Spectra evolution of non-coated, GO-coated, and IgG-immobilized dLPG. Reproduced from [93] with permission of Elsevier.

Long-period fiber gratings are also called transmission fiber gratings, and their period is relatively long, which is generally hundreds of microns. In 2017, Liu Chen’s research team studied biosensors based on graphene oxide nanosheets that functionalized dual-peak long-period grating (dLPG), and proposed a label-free biosensor based on GO-coated dLPG for the real-time detection of the biological affinity between the antibody and the antigen; this was through a new GO deposition technology that combines chemical bonding and physical adsorption, enabling GO to bind to dLPG firmlyand uniformly with good stability and high repeatability. Figure 5a shows the schematic diagram of the optical fiber biosensor containing the dLPG coated with the graphene oxide-linking layer; Figure 5b shows that the Raman spectrum of the GO-coated fiber and bare fiber. Compared with the bare fiber, the Raman spectrum of the GO-coated fiber appears as a red curve consisting of three prominent peaks, which proves the addition of GO successfully. Figure 5c plots the evolution of the RI transmission spectrum of bare dLPG at different sucrose concentrations, and the dual-peak moving in opposite directions as the solar reflectance index (SRI) increases. As shown in Figure 5d, the dependence of dual-peak separation against the change of the SRI demonstrates a non-linear relationship. Figure 5e plots the evolution of the transmission spectrum of GO-dLPG at different sucrose concentrations, and the intensity of the GO-dLPG dual-peak decreases significantly as the SRI increases. The GO deposition enhances the light matter interaction between the cladding and the surrounding medium, thereby improving the RI sensitivity. Figure 5f depicts the transmission spectra captured in DI water before and after GO deposition. After IgG immobilization, the separation and intensity of the dual-peak increased relative to both the GO deposition and IgG immobilization procedures. Through characterization and testing, the RI sensitivity of dLPG was improved by 200% and 155% in low RI (1.333–1.347) and high RI (1.430–1.441) regions, respectively. The detection limit is 7 ng/mL, which is 10 times that of the uncoated dLPG biosensor and 100 times that of the LPG immunosensor. The GO-dLPG biosensor has the advantages of label-free, real-time monitoring, ultra-high sensitivity and multi-purpose, and provides a good biological analysis platform for biosensing [93].

**Figure 6:**
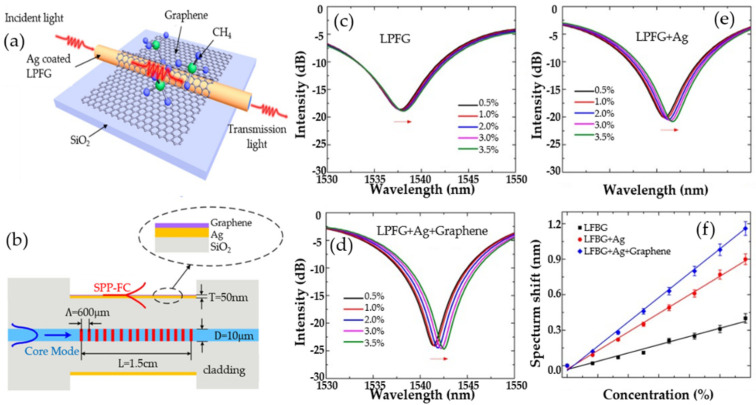
(**a**) The structure diagram of the graphene-based LPFG SPR sensor. (**b**) The sectional diagram of the graphene-based LPFG SPR sensor. (**c**) Transmission spectra of LFBG sensor. (**d**) Transmission spectra of graphene-based LFBG SPR sensor in different concentrations of methane gas. (**e**) Transmission spectra of Ag-coated LFBG SPR sensor. (**f**) Resonance wavelength shift versus concentration of methane. Reproduced from [94] with permission of the Multidisciplinary Digital Publishing Institute.

In 2017, Wei Wei’s research team proposed a SPR sensor based on graphene LPG for high-sensitivity gas-sensing detection. A layer of single-molecule graphene is coated on the surface of the Ag film of the long-period optical fiber SPR sensor to enhance the evanescent electric field intensity on the surface of the optical fiber, thereby enhancing the interaction between the SPR wave and the molecule. Figure 6a shows the structure diagram of the proposed graphene-based LPFG SPR sensor. Its longitudinal section is shown in Figure 6b (SPP-FC refers to surface plasmon polariton–fiber cladding). Figure 6c–e show the transmission spectra of three sensors that were directly exposed to different concentrations of methane to evaluate the sensitivity. As the methane concentration increases, the resonance spectra of the sensors all exhibit a wavelength red shift. Figure 6f shows the linear fitted curves of the Figure 6c–e, which exhibits good linearity and indicates that the graphene-based LPFG SPR sensor has the best sensing performance. The experimental results demonstrate that the graphene-based long-period fiber grating SPR sensor has a sensitivity of 0.344 nm%^−1^ to the change of methane gas concentration, which is 2.96 times and 1.31 times higher than the traditional long-period fiber-grating sensor and the silver-plated long-period fiber-grating SPR sensor, respectively. The performance of the LPFG SPR sensor can be improved by covering the graphene. It has good response characteristics and repeatability, which provides a broad prospect for high-sensitivity graphene gas sensors [94].

In 2020, Wang’s research team developed a graphene oxide micro-tapered long-period fiber-grating (MTLPG) biosensor for the detection of hemoglobin molecules [95]. The resonance wavelength is shifted when the hemoglobin molecule is adsorbed to the fiber surface, allowing the hemoglobin concentration to be detected. The sensitivity of detecting human hemoglobin in deionized water, urea, and glucose is 2 nm/(mg/mL), 1.03 nm/(mg/mL), and 0.73 nm/(mg/mL), respectively. The detection limit is as low as 0.02 mg/mL, which is 48 times lower than that of the uncoated MTLPG biosensor and well below the defined anemia hemoglobin threshold by the World Health Organization. The biosensor has strong sensitivity, high efficiency, the characteristics of label-free, real-time monitoring, stability, reusability, and so on, which can be applied in the fields of biomedicine and biochemistry.

In 2021, Esposito’s research team developed a long-period fiber-grating biosensor based on the graphene oxide double-clad structure for the detection of C-reactive protein (CRP) in human serum [96]. The minimum detection limit for the determination of CRP in serum is 0.15 ng/mL, covering the clinical application range of 1 ng/mL–100 μg/mL. This is one of the lowest values in the literature reports on optical fiber biochemical sensors thus far, and it is also the direction we need to continue to explore in the future.

#### 4.1.2. Graphene Bragg Fiber-Grating Sensor

Bragg fiber gratings are also called reflective or short-period fiber gratings, and the grating period is only a few hundred nanometers. In 2014, Sridevi et al. developed a graphene oxide-etched Bragg fiber-grating biosensor for the detection of the protein concanavalin A (Con A) [97]. In 2015, Yao et al. developed a graphene-based D-type Bragg fiber-grating biosensor to accurately detect the concentration of red blood cell solution [98]. Due to the addition of graphene, the sensor demonstrates high sensitivity for detecting surrounding biochemical parameters with a value higher than 1 pm/ppm. This biochemical sensor has a compact structure, which is clinically acceptable and provides good recoverability, providing an advanced sensing platform for high sensitivity in situ and live cell detection applications.

**Figure 7:**
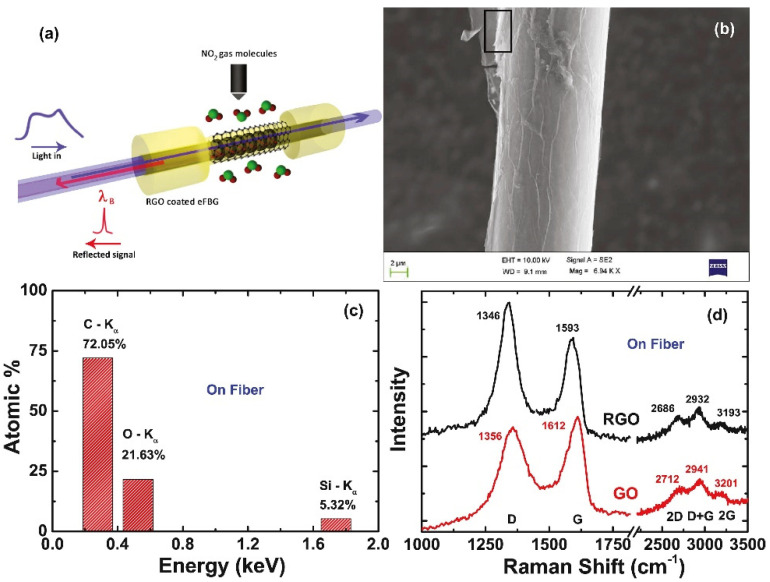
(**a**) Schematic illustration of the NO_2_ gas-sensing mechanism on the RGO-coated eFBG. (**b**) SEM image of the eFBG sensor showing uniformly coated RGO in the FBG region. (**c**) EDAX spectra recorded from RGO flakes (designated by the black-colored rectangular box in Figure 6a) coated on the eFBG sensor surface. (**d**) Raman spectra of GO flakes (coated on eFBG sensor), before and after the reduction. Reproduced from [99] with permission of Elsevier.

In 2016, the Sridevi research team studied an etched fiber Bragg grating (eFBG) chemical sensor based on the redox graphene (RGO) coating for NO_2_ gas in room temperature sensing. Figure 7a shows the schematic of NO_2_ gas-sensing mechanism on RGO coated eFBG. Figure 7b shows the SEM image of eFBG sensor coated with RGO prepared by the reduction of the GO coated on the eFBG surface using hydrazine. Figure 7c shows the EDAX spectrum of RGO on the eFBG sensor. From the EDAX data, the atomic ratio of carbon to oxygen is found to be 3.5 after reduction, which corroborates the reduction of GO to RGO. Figure 7d shows the Raman spectrum before and after the reduction of GO flakes coated on the eFBG sensor. The peak position of the 2D band is similar to that of a monolayer graphene prepared using mechanical exfoliation. The detection limit is 0.5 ppm and the sensitivity is 0.8 pm/min. The adsorption and desorption of NO_2_ gas molecules on RGO changes the refractive index by increasing and decreasing the local carrier concentration. The RGO-coated eFBG sensor can detect sub-ppm level NO_2_ gas. By using multiple gratings in the same fiber, the performance of the sensor can be improved to sub-ppb level, and the response time can be shortened, providing more effective biochemical-sensing reference data [99].

### 4.2. Graphene No-Core Fiber Sensor

The classic approach of improving the sensitivity of optical fiber sensors involve corroding the structure of fiber cladding [100]. However, this etching method is difficult to precisely control. This will not only increase the surface roughness of the fabricated sensing fiber, but also reduce the mechanical strength of the fiber, which is not conducive to subsequent fusion and sensing applications. Moreover, researchers began to ponder the use of surface plasmon resonance (SPR) method for optical fiber-sensing for solving the above problems. The performance of the sensor can be improved by the SPR method with localized surface enhancement effect, but the plasmonic sensor needs to introduce a metal with a negative dielectric constant as the excitation condition, which will greatly increase the internal loss of the sensor and affect the sensitivity and quality factor of the sensor. The single-multi-single-mode (SMS) sensors based on the step refractive index distribution can achieve multi-mode interference. Replacing multimode fiber with coreless fiber (NCF) can greatly overcome the above-mentioned shortcomings caused by chemical corrosion, and improve the sensitivity, and repeatability at the same time.

**Figure 8:**
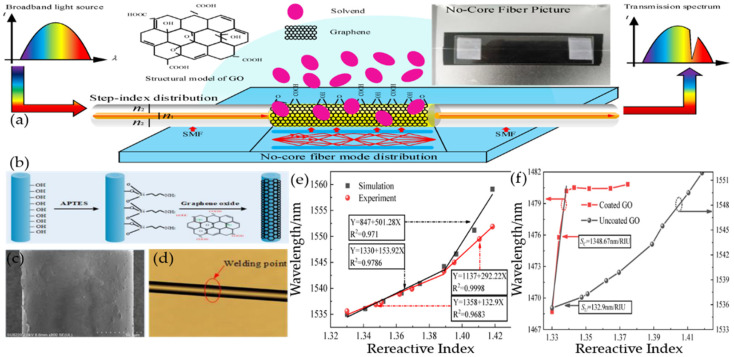
(**a**) Schematic diagram of the GO-sensitized SNS sensor. (**b**) Flow chart of GO production (**c**) SEM image of GO-coated fiber sensor. (**d**) Micrograph of solder joint. (**e**) Sensitivity and linearity at different RI wavelengths (simulated and experimental). (**f**) The relationship between the characteristic wavelength and the refractive index of the coated and uncoated GO. Reproduced from [101] with permission of the Multidisciplinary Digital Publishing Institute.

Yang HongYan’s research team designed a single-mode no-core single-mode (SNS) fiber step-index distribution sucrose sensor for glucose detection. Figure 8a shows the structure of the GO-sensitized SNS sensor. Figure 8b is the fabrication flow diagram. Figure 8c is the NCF coated with GO. Figure 8d is a photomicrograph of the solder joint. As the diameter of the NCF is the same as that of the single-mode fiber, the three segments of the fiber can be perfectly coupled after fusion. Figure 8e shows the sensitivity and linearity of the wavelength as a function of RI in both simulations and experiments. The results demonstrate that the sensor has higher sensitivity to higher RI solutions, and the actual sensitivity measured experimentally is lower than the theoretical sensitivity of the simulation. Figure 8f shows the relationship between the characteristic wavelengths and the RI of the coated GO and uncoated GO. The experiments demonstrate that the method of coated GO cannot be used for the RI measurement when the RI is higher than 1.3385, but in the low RI range of 1.3 to 1.3385, the sensitivity of the GO-coated SNS sensor can be improved by nearly 914.8%. Due to the -OH and -COOH groups, the graphene oxide surface will react with sucrose’s -OH to form a hydrogen bond, which causes sucrose adsorption when the graphene surface is oxidized. The adsorption reaches saturation rapidly and the corresponding characteristic wavelength changes little in high-concentration sucrose solution. Consequently, it is possible to improve the sensing field of the sensor through these excellent properties. This research provides a novel method for us to detect sucrose solution [101].

### 4.3. Graphene Photonic Crystal Fiber Sensor

Photonic crystal fiber (PCF) has been proven to be a good prism. Single-mode transmission, excellent birefringence, and tunable dispersion characteristics are all advantages of its unique structure and flexible design [102]. The combined action of graphene and metal on PCF has been proven in studies to increase the performance of traditional PCF sensors. At present, graphene photonic crystal fiber biochemical sensors based on plasmon resonance have been well researched, providing a new path for graphene-based fiber biochemical sensors.

In 2014, Dash et al. proposed a birefringent PCF-SPR biochemical sensor based on a graphene-silver layer [80]; graphene helps prevent the oxidation of silver used as a plasma active metal, thereby improving the sensitivity of the sensor that is higher than the widely used bimetal configuration. In 2017, Yang et al. proposed a PCF-SPR biochemical sensor based on a graphene-silver layer with a wavelength sensitivity of 2520 nmRIU^−1^ and a resolution of 3.97 × 10^−5^ RIU [103]. In the same year, Wang et al. proposed a dual-core PCF-SPR biochemical sensor based on a graphene-silver layer, which has an average sensitivity of 4350 nmRIU^−1^ in the refractive index range of 1.39–1.42 and the maximum sensitivity reaches 10,000 nmRIU^−1^; the resolution is 1 × 10^−6^ RIU in the range of 1.43–1.46 [104].

Rifat et al. coated graphene copper on the outer surface of PCF in 2016, and constructed a PCF-SPR biochemical sensor based on the graphenecopper layer [105]. Within the refractive index range of 1.33–1.37, the amplitude sensitivity is 140 RIU^−1^, resolution is 7.1 × 10^−5^ RIU, wavelength sensitivity is 2000 nmRIU^−1^, and resolution is 5 × 10^−5^ RIU. Lou et al. proposed an eccentric PCF-SPR biochemical sensor based on graphene-gold coated on the outer surface of PCF in 2019 [106]. After graphene is added, the average sensitivity of the sensor increases from 2938.86 to 5171.51 nmRIU^−1^; the maximum sensitivity is 8600 nmRIU^−1^.

**Figure 9:**
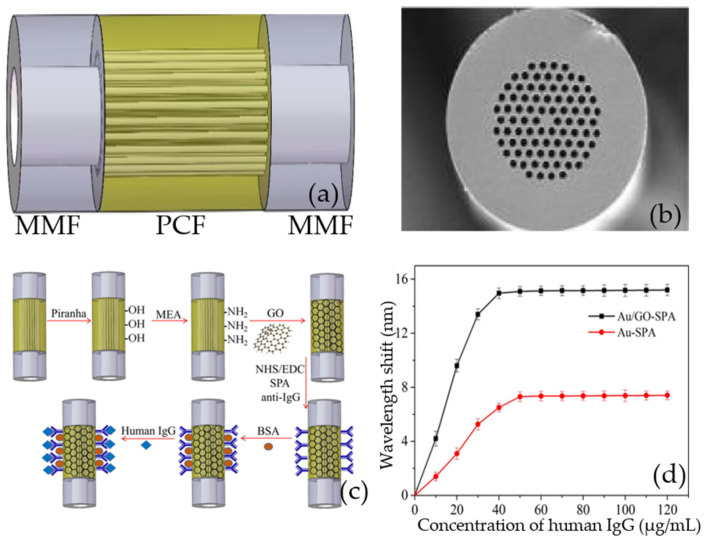
(**a**) The schematic diagram of MMF-PCF-MMF (multimode fiber-photonic crystal fiber- multimode fiber) sensor. (**b**) The end-face micrograph of PCF. (**c**) GO and SPA modified process for IgG immunoassay. (**d**) Goat anti-human IgG immobilized on the sensor surface. Error bars represent the standard deviation of three independent experiments. Reproduced from [107] with permission of Elsevier.

In 2018, Wang qi’s research team proposed and developed a sensitivity-enhanced SPR biochemical sensor based on the GO complex and staphylococcal protein A (SPA) co-modified PCF for detecting human IgG. [107]. The schematic diagram of the photonic crystal fiber sensor is shown in Figure 9a, and the end-face microscope image of the photonic crystal fiber is shown in Figure 9b. Figure 9c shows graphene oxide and SPA modification method for the IgG immunoassay. Figure 9d shows that the wavelength shift curves of goat anti-human IgG immobilized on the surface of the Au/GO-SPA and Au-SPA sensors. The experimental results suggest that the sensor has not only a low temperature effect, which can eliminate the temperature interference in the experiment, but is also specific to human IgG and has good performance stability. The addition of graphene oxide greatly improves the sensitivity of the photonic crystal fiber, and has great application potential in the immunoassay.

Compared with circular-shaped PCF, D-shaped PCF has an asymmetric cross-sectional structure and a wide and flat side polishing surface, which is not only conducive to the transfer and deposition of graphene, but also make it easy to reprocess the graphene on the side-polishing surface [108]. Chemical etching, laser etching, and side polishing can be used to create D-shaped PCF from regular PCF. In recent years, researchers have been working on graphene D-shaped photonic crystal fiber biochemical sensors based on surface plasmon resonance; graphene/metal film/polymer composite materials have been employed to adjust the side-polishing surface to enhance the sensitivity of the sensor.

In 2018, Tong Kai’s research team proposed a D-shaped PCF biosensor based on SPR graphene coating [109]. Graphene is coated between the Ag layer and D-PCF. The biosensor has a refractive index range of 1.34–1.40, an average sensitivity of 4850 nmRIU^−1^, and a resolution of 2 × 10^−5^ RIU. The sensor can be applied to real-time detection of biomolecules and tiny drug molecules due to the long-distance transmission, high sensitivity and resolution.

Yashar et al. proposed a D-shaped PCF biochemical sensor based on SPR in 2021 [110]. They studied the combination of three materials (silver, silver-graphite, and silver-graphene) as the plasmonic nano-film of SPR-PCF, and the results demonstrated that graphene-assisted PCF has the best comprehensive performance, with an average sensitivity of 2320 nm RIU^−1^ and a maximum amplitude sensitivity of 192 RIU^−1^ in the refractive index range of 1.27–1.37 in the internal filling detection scheme; the maximum sensitivity is 22,400 nmRIU^−1^ and the maximum quality factor (FOM) is 127 in the external-filling detection scheme.

Instead of using metal layers as plasma materials, some researchers employed two-dimensional materials and put a layer of graphene on the film for study. In 2021, Tian et al. proposed a graphene-coated D-shaped PCF chemical sensor based on SPR, which uses indium tin oxide (ITO) instead of metal as the plasma material [107]. The sensor has a wavelength sensitivity of 12,000 nmRIU^−1^ and a maximum resolution of 8.33 × 10^−6^ RIU in the refractive index range of 1.21–1.32. This type of chemical sensor offers good sensing performance and a lot of economic promise in biochemical sensing and environmental monitoring and assessment. However, it cannot obtain excellent sensing performance in the entire effective sensing range which should be the direction of our future work.

## 5. Conclusions

For the past few years, graphene-based fiber-optic biochemical sensors have demonstrated excellent performance in genetic engineering, medical diagnosis, environmental monitoring, and food safety, etc. However, the widespread application of graphene-based commercial fiber biochemical sensors still faces challenges, and the following key issues need to be solved: Firstly, the current research on graphene is still limited to theories and laboratories-the existing preparation methods of graphene are not perfect enough. How to prepare high-quality graphene with controllable size and thickness, no defect, no pollution, and uniform distribution at a low cost is one of the urgent problems to be solved. Secondly, when graphene is transferred to the surface of the fiber, it is easy to be contaminated and damaged. How to realize the lossless and high-quality coupling between graphene and fiber is the second key problem to be solved. Thirdly, graphene’s flexible photoelectric characteristics make it difficult to find a perfect solution to the cross-sensitive detection. For example, temperature can interfere with the gas sensor when measuring gas concentrations. Finally, the repeatability and stability of graphene sensors are not optimistic either. When the external environment causes the electronic structure of graphene to change, it will result in graphene taking a certain time to recover. It is difficult to automatically restore it to the original state, which leads to the poisoning of the sensor when graphene adsorbs chemical molecules.

As the world is suffering from COVID-19, the public health security system of mankind is facing unprecedented challenges. If the research of the graphene fiber biochemical sensor can break through the limitation of theory and experiment and put it in to large-scale industrial production, it will rapidly improve the pace of human intelligent life. Here, based on the above reasons, by choosing three graphene-based fiber biochemical sensors with special fiber structures (namely fiber gratings, coreless fibers and photonic crystal fibers) as breakthrough points, we reviewed the new research development and existing problems of the graphene-based fiber biochemical sensor in detail, which provides reliable data and novel ideas for the practical application of the graphene-based fiber biochemical sensor in order to achieve a major breakthrough in the fiber biochemical sensor. 

Although much work still need to be conducted for the application of the graphene-based fiber biochemical sensor, the future for graphene-sensing devices appears very exciting. In order to improve the performance of graphene-based fiber-optic biosensors, there have been many improved progresses in the preparation of graphene, the structural form of graphene and the design of graphene-based fiber-optic biosensors. For example, functionalized graphene is modified by other molecules or atoms, such as doping molecules and adding substrates, etc., in order to improve the performance of graphene. So far, in addition to graphene and its derivatives, there have been many reports on novel two-dimensional materials, such as molybdenum disulfide (MoS_2_) and black phosphorus (BP), etc., which is the multifunctional integration with optical fiber to realize biochemical sensing. Moreover, graphene, as a modifier combined with semiconductor materials, in order to realize the application of graphene photoelectric sensors and graphene-based flexible implantable biochemical sensors, is mushrooming development. In the future, graphene will present extraordinary talents in the field of artificial intelligence and wearable sensing as a new material for fiber sensors.
